# PReS-FINAL-2195: The comparison of the efficacy of once and twice daily dosage of colchicine in pediatric patients with Familial Mediterranean Fever

**DOI:** 10.1186/1546-0096-11-S2-P185

**Published:** 2013-12-05

**Authors:** A Polat, C Acikel, B Sozeri, I Dursun, O Kasapcopur, H Peru, I Dokurel, H Poyrazoglu, S Bakkaloglu, A Delibas, Z Ekinci, NA Ayaz, Y Kandur, E Unsal, B Makay, F Gok, S Ozen, E Demirkaya

**Affiliations:** 1 FMF Arthritis Vasculitis and Orphan Disease Research in Paediatric Rheumatology (FAVOR), Ankara, Turkey

## Introduction

Familial Mediterranean Fever (FMF) is the most common periodic fever syndrome, characterized by recurrent fever and serositis attacks. Colchicine is the treatment of choice. Colchicine is given in two or three divided doses due to its side effects. It has been stated that colchicine unresponsive patients are in fact non-compliers because there is an inverse relationship between daily dosage and patient compliance.

## Objectives

In this study, it was aimed to investigate whether the once daily dosage would be as effective as twice daily dosage regimen in reducing disease symptoms and inflammation both in clinical and laboratory parameters addition to manifesting with similar side effects.

## Methods

Seventy-nine patients between 5 and 16 years of age with a clinical and genetically confirmed diagnosis of FMF and who had never received colchicine were included in the study. The drug was started in one or two doses randomly. A total of 42 patients received colchicine in once daily dosage while 37 patients had in twice daily dosage. The clinical, laboratory parameters and drug side effects were assessed on three clinical visits two months apart.

## Results

Mean age was 7,9 ± 1,9 and 7,7 ± 2,1 years and female/male ratio was 21/21 and 19/18 in one and two-dose groups respectively. There were 22 patients with heterozygote and 57 homozygote MEFV mutation in our study cohort. After colchicine treatment all patients demonstrated improvement in FMF clinical features and Mor and Pras scores. There were no difference between one and two-dose groups about these improvements in clinical features and decreasing in Mor (*p *= 0,555) and Pras (*p *= 0,236) scores. After colchicine treatment started the levels of erythrocyte sedimentation rate (ESR), C reactive protein (CRP), serum amyloid A (SAA), and S100A12 all decreased. No difference was observed between one and two-dose groups when compared the changes in the levels of ESR (p = 0,462), CRP (p = 0,385), SAA (*p *= 0,761) and S100A12 protein (*p *= 0,944). Patients in both groups manifested with side effects of colchicine after the treatment. Although diarrhea seemed to be more frequent among one-dose group, no significant difference was present about side effects of the drug between one and two-dose groups.

## Conclusion

The results indicated that there was no difference between once and twice dose groups considering improvements in clinical and laboratory findings. Drug side effects were similar in both groups; diarrhea was frequent in one-dose group. Once daily dosage is as effective as twice daily dosage scheme and can be prescribed considering the tolerance of a patient.

## Disclosure of interest

None declared.

